# Exploring Young Adults' Experiences of Atopic Dermatitis Self‐Management and Use of Community Pharmacy: A Qualitative Study

**DOI:** 10.1111/hex.70378

**Published:** 2025-08-07

**Authors:** Abdi Berk Cayci, Adam Pattison Rathbone, Charlotte Lucy Richardson, Laura Lindsey

**Affiliations:** ^1^ School of Pharmacy, Faculty of Medical Sciences Newcastle University Newcastle upon Tyne UK; ^2^ Department of Clinical Pharmacy, Faculty of Pharmacy Hacettepe University Ankara Türkiye; ^3^ Great North Children's Hospital Newcastle upon Tyne NHS Foundation Trust Newcastle upon Tyne UK

**Keywords:** atopic dermatitis, community pharmacists, eczema, pharmacy, self‐management, young adults, youth

## Abstract

**Introduction:**

Atopic dermatitis (AD) is a long‐term skin condition. Transitioning from parent‐dependant management to independent self‐management, means young adults may experience fluctuations in their care during transition period. However, little is known about the impact this transition has and support young adults receive. From 2026, community pharmacists (CPs) in the UK will be prescribers, a potential source of support for AD management for young adults. This study aimed to explore young adults' experiences of transitioning to self‐manage AD with particular emphasis on accessing CP support in self‐management.

**Methods:**

One‐to‐one, semi‐structured interviews were conducted with 25 young adults aged 18–24 years with AD in the UK. Data was analysed with iterative, inductive thematic analysis. Ethical approval was received before data collection.

**Results:**

Three themes were identified: (i) *journey to self‐management* which shows that transition to self‐management came with diverse and unique challenges, (ii) *impact of concerns about AD and its treatments on self‐management* where participants reported concerns about AD symptoms and treatments affecting self‐management, (iii) *limited use of pharmacy for health advice*, in which young adults described poor awareness of CPs as a source of information but as access to prescribed treatment, due to challenges seeing specialists.

**Conclusions:**

Young adults with AD have unique needs and concerns during transition to self‐management. Young adults perceived CPs being able to prescribe could support continued access to prescribed treatments, but did not currently consider CPs as a source of advice. Further work is needed by policymakers and practitioners to improve awareness of CPs' skills and roles among young adults, which may help their concerns to be addressed in community pharmacies.

**Patient or Public Contribution:**

Lived experiences have been central to the development and design of this study. Listening to a family member's experiences of AD inspired the lead author to research young adults' AD management. The data collection process was informed by two people with AD whose advice was sought on how best to approach the topic with young adults and on the topic guide. Changes to wording were made in response to the comments from the two people with AD. Further, after each interview, each young adult participant was asked to give their feedback on the interview guide and how it could be further improved. All participants were happy with the questions and one participant suggested an additional question that had not been included in the topic guide previously. To help the interpretation of study findings informal discussions were conducted with young adults with and without AD. These young adults were not study participants.

## 1 Introduction

Atopic dermatitis (AD), also known as eczema, is a chronic skin condition, characterised by itchy, erythematous, and inflammatory lesions [1, 2]. Typically, AD presents in infancy and childhood, accounting for around four‐fifths of AD cases [3]. It persists into adulthood with the prevalence ranging between 2.1% and 4.9% [4]. Since there is no definitive cure for AD, the goal of treatment is to keep AD controlled [5]. AD varies in severity ranging from mild to severe including moderate cases and a stepwise treatment is recommended depending on severity of the condition, with emollients considered as a cornerstone of treatment [6]. Topical corticosteroids (TCs) are first‐line treatment to manage AD flare‐ups, and topical calcineurin inhibitors may be used as second‐line anti‐inflammatory treatment [6, 7]. According to a recent clinical guidance by the European Task Force on Atopic Dermatitis, TCIs, such as tacrolimus, are also recommended as first‐line treatment option to manage moderate and severe AD on sensitive body areas, such as eyelids and genital areas, for long‐term use [6].

As the condition persists from childhood through to adulthood, it is important to consider the transition of self‐care from care primarily managed by parents, to one led by the patient as an independent adult [8]. Self‐management is a concept describing how patients manage chronic conditions and includes some key components, such as prevention, having a care plan and managing the effect of the condition [9]. AD can continue to impact an adult's quality of life negatively [10], and so exploring young adults' perspectives of AD self‐management from paediatric to adult care may help to identify challenges to treatment continuation. Young adults are defined as people aged between 18 and 25 years. This group of people has their specific needs in terms of healthcare, which should be researched separately from younger and older people [11]. It is known young people need tailored knowledge and support when adjusting to AD self‐management [8, 12]; however, this study involved both young adults and younger people, thus not focusing on the unique experiences of young adults as a patient group nor reporting findings solely for this group. In addition, previous work has focused on adults, young people and parents of children with AD, not young adults specifically [13]. Therefore, further research is needed to identify the impact of the transition to self‐management on young adults.

Healthcare professionals (HCPs) have a key role in supporting self‐management though some young adults may struggle to access healthcare, for example, when young adults' try to access smoking cessation or sexual health services [14, 15, 16]. These services, typically offered by community pharmacies, may mean young adults also struggle to access care for other conditions, including AD [17]. Community pharmacists (CPs) can be a source of advice and support for range of health conditions, but are often overlooked and underutilised by patients [18]. In addition, previous work shows limited awareness of the skills of CPs for patients with skin conditions [19], but there is a gap in the literature around how young adults with AD perceive CP support and seek help from CPs within their self‐management. Therefore, this study aimed to explore young adults' experiences of transitioning to self‐manage AD with particular attention to accessing support from CPs within their self‐management.

## 2 Methods

### 2.1 Study Design and Participants

A qualitative study using one‐to‐one semi‐structured interviews was conducted with young adults with AD. The term young adult is used to refer to study participants aged 18–24 years old [11]. Young adults were eligible to participate if they were (i) aged between 18 and 24 years old, (ii) diagnosed and prescribed any medicine for AD, and (iii) resident in the UK. Participants were recruited through a public advertisement campaign including posters in the local area, including across university campus, colleges, cafes, and retail outlets, additionally online through youth organisations, forums and blogs. Eligible participants were screened using purposive sampling approach to capture a wide range of experiences. The aim was to recruit participants of different ages, ethnic groups and AD severities. Before the interview, participants were sent a participation information sheet and consent form. Ethical approval was granted by the University Ethics Committee (reference number: 22623/2022). This study is reported in line with recommendations by the Standards for Reporting Qualitative Study (see Appendix) [20].

### 2.2 Reflexivity

To increase transparency and trustworthiness, the authors reflected on their identities and how this could influence research findings [21, 22]. The research team included three pharmacists and a psychologist. Three authors were experienced qualitative researchers in social and cognitive science, health services research, and research with young adults, and were supervising one author's doctoral studies. Two authors had experience of treating patients with AD in community pharmacy settings in the UK. During data collection and analysis, the authors used their unique knowledge and experiences to interpret data. Importantly, during data collection, where participants disclosed or requested information relating to their care, they were signposted to other services, to prevent clinical interactions from biasing research interactions [21]. Participants and researchers did not have an existing relationship, and researchers did not influence the participants' care in any way.

### 2.3 Data Collection

As AD severity varies in patients, which impacts treatment decisions and AD management, participants completed a Patient Oriented Eczema Measure (POEM) questionnaire, a validated self‐reporting tool used to measure AD severity, before the interview [23, 24]. In person or online interviews were conducted between February and November 2023. An interview topic guide (see supporting material) consisting of open‐ended questions covering AD management (diagnosis, treatment, AD experience) and interaction with CPs, was used. The topic guide was developed and piloted with two people with AD, who were not recruited as participants. All interviews were audio recorded, transcribed verbatim and anonymised by one author (ABC). Participants were given pseudonyms based on their order of participation.

### 2.4 Data Analysis

Data analysis was conducted using NVivo (version 1.6.1) and Braun and Clarke's six‐phase inductive thematic analysis process [25]. Transcripts were reread several times to identify initial ideas, then transcripts were coded line by line by one author (ABC). After initial coding, a coding manual was produced and discussed by two authors (ABC, LL). During coding, preceding transcripts were revisited and ameliorated to provide relevance while new codes emerged. Codes were assessed and any discrepancies were resolved by all authors. Thereafter, codes were categorised to identify preliminary themes by one author (ABC). Preliminary themes were reviewed by all authors and consensus was reached on theme. Each theme was defined, ensuring it encompassed a centralised concept which gives shared meaning within a theme [26]. As the last two interviews did not add any new codes and further dimensions to an existing code, it was agreed that data saturation was reached, and data collection was stopped [27]. Regarding the POEM questionnaire, each participant score was a self‐reported measure based on their answers to the frequency of seven common AD symptoms on a 5‐point Likert scale, ranging from ‘clear/almost clear’ to ‘very severe’ [23, 24].

## 3 Results

In total, 25 participants were recruited and interviewed (see Table 1 for a demographic summary). Most participants were female (*n* = 20). The range of ethnicities was diverse with two highest groups being: white/white British (*n* = 9) and Asian/Asian British (*n* = 8). Most participants had moderate AD (*n* = 16) with four mild and five severe cases also being included. Interviews lasted 38 min on average. Three key themes were identified: (i) journey to self‐management, (ii) impact of concerns about AD and its treatments on self‐management, and (iii) limited use of pharmacy for health advice.

**TABLE 1:** Demographics of participants interviewed in this study.

	Participants (*n* = 25)
Age, years	
Median age (range)	20.92 (18–24)
Gender	*n* (%)^a^
Female	20 (80)
Male	4 (16)
Nonbinary	1 (4)
Occupation	
Student	21 (84)
Full‐time employee	4 (16)
Ethnicity	
White/White British	9 (36)
Asian/Asian British	8 (32)
Black/Black British	3 (12)
Mixed ethnic groups	2 (8)
Other ethnic groups^b^	3 (12)
The onset of AD	
Infancy	10 (40)
Childhood	12 (48)
Adolescence	3 (12)
Severity of AD via POEM^c^	
Mild	4 (16)
Moderate	16 (64)
Severe	5 (20)
Duration of interviews	
Mean duration (range)	38 min (21–54 min)

^a^ All figures are presented as *n* (%) unless otherwise stated.

^b^ Other ethnic groups include ethnics other than White/White British, Asian/Asian British, Black/Black British.

^c^ Mild AD is shown between 3 and 7, moderate between 8 and 16, severe between 17 and 24 in POEM bandings.

### 3.1 Journey to Self‐Management

This theme encompasses participants' experiences on their transition to self‐management as a young adult, and how this affects their AD management.

For participants, previous parental inclusion was perceived as positive in their childhood. Parental influence continued to remain strong for many participants although they lived independently from parents. Most participants had started becoming proactive about their self‐management and were aware of the responsibility of their AD treatment once they were 18 years old, with some participants starting this transition earlier.

‘… when I was 18 and came to [university] and I had to manage myself like even things like buying, paying for my prescriptions and buying my creams and things myself. I've had to start doing and being more proactive’.

P22 (female, 22‐year‐old, severe AD)

Once they were more proactive in their self‐management, some participants reported observing improvements in AD symptoms, with less severe flare‐ups. Most participants felt they were more adherent to treatments now, though a few reported discontinuing medicines after their symptoms improved.

Participants shared they had learnt more about AD talking to friends with similar experiences and trusted their treatment suggestions, contributing to their self‐management and well‐being. Participants also sought advice about AD management on social media, but recognised a need to use reliable sources, such as health services websites. However, one participant approached some health information websites suspiciously.

‘I don't even know if [a health information website] is really accurate, but it has like sources underneath, reviewed it, but I don't know. I take that with more of a pinch of salt’.

P23 (male, 20‐year‐old, moderate AD)

Participants had experienced challenges with self‐management. Several participants welcomed living independently because of family expectations on treatment causing pressure. However, some participants who had left home identified emotional factors, such as stress and anxiety, as underlying reason for the exacerbation of AD. In addition, many participants shared that AD could flare up quickly and described their experience as a ‘roller coaster’, which negatively influenced their mental well‐being.

‘So sometimes [fluctuating flare‐ups] really affects my mental health so badly because I get angry at myself for no reason. I get angry at my [AD]’.

P21 (female, 19‐year‐old, moderate AD)

Participants reported recognising environmental triggers, such as allergies, as they started to self‐manage AD. This included identifying carpets in accommodation, the hardness of tap water, doing laundry, the weather and climate as triggers. Some participants adjusted their lifestyle to control triggers, such as not scratching, using micellar water and nonbiological detergents. Participants also mentioned experimenting with elimination diets to detect triggering foods. They had felt disappointed after stopping certain foods; however, they reported experiencing a compulsion to make these changes to control their AD.

‘… but if [the food] specifically contains [egg], I don't have it … I just avoid’.

P14 (female, 22‐year‐old, moderate AD)

Economic factors affected young adults' self‐management. Some participants perceived the cost of the medicines as an ‘extra’ on their budget. For overseas students, finding an equivalent medicine to one prescribed after leaving home was another problem. A couple of participants also experienced prescribed medicines becoming unavailable and were replaced with other medicines, which they did not think worked as well. This led to out‐of‐pocket purchases of the original treatment.

‘[Specialists] stopped giving me [original treatment] and gave me something else, which didn't work. So I again started buying [original treatment]’.

P2 (female, 20‐year‐old, moderate AD)

Participants' experiences of the transition to adulthood included acknowledging that AD was a vicious cycle which would not fully go away. Participants described living with AD as a learning curve.

‘[AD] goes away and then about 2 weeks later it starts up again. It's just a constant cycle. It's kind of learning how to live with [AD]’.

P15 (female, 21‐year‐old, severe AD)

Collectively this finding demonstrates participants' experiences of learning how to manage their AD symptoms was a journey, becoming familiar with using information sources, including social media, peers, and other health information, identifying triggers, and managing access to medications and adapting their newly independent lifestyles to manage symptoms.

### 3.2 Impact of Concerns about AD and Its Treatments on Self‐Management

This theme describes participants' concerns about AD and its treatment patterns, and how these concerns have impacted their self‐management. Itchiness and pain were the most common symptoms that participants wished to eliminate. Participants expressed strong desires to be completely clear of AD.

‘I'd like to be free of [AD]. . My friends have said to me “If there was £1 million on one side and get rid of eczema on the other, which would you choose?” and I always said “get rid of my [AD]”’.

P15 (female, 21‐year‐old, severe AD)

Participants described having visible signs of AD were sources of embarrassment and received negative comments from peers, such as being compared to crocodile skin. Experiences of stigma, ‘bullying’ and social isolation were common.

‘…can be isolating, especially when [stigma]'s been something that you have been bullied about and it's something that has already made you retreat into yourself’.

P19 (female, 22‐year‐old, severe AD)

Concerns about their visual appearance and AD mostly started during adolescence, and this concern remained during the transition to independence. Some participants were keen to conceal AD symptoms, particularly on facial areas, as the visibility of these symptoms impacted self‐confidence during the transitional period. Participants reported that the physical and mental impact of AD were underestimated and not well understood by society, including family members, friends, and HCPs alike.

Participants were concerned about the use of TCs in their self‐management due to skin penetration and side effects of TCs. Some participants also reported concern about malignancy risk after TCI use. Participants' most common concern was TC withdrawal syndrome, influenced by the use of social media, which young adults referred to as a source for getting information during transition to self‐management.

‘I'm really like a bit paranoid about [TC] withdrawal syndrome and I think that's definitely an impact of social media’.

P15 (female, 21‐year‐old, severe AD)

Consequently, some participants reported using TCs sparingly and only for short‐term because of the concerns about the use of TCs including withdrawal concern. Additionally, a minority of participants were aware of fingertip units (FTUs), but they had never made use of it to apply TC. Some participants highlighted never getting information about FTU from HCPs including CPs. In a complete contrast, a few participants acknowledged overusing TCs, particularly one using on her face to alleviate AD symptoms.

‘I've used [TCs] like on my face when it shouldn't have been used… I used higher strength until it calmed down’.

P16 (female, 23‐year‐old, moderate AD)

Collectively, these findings demonstrate young adults' lived experiences of AD including treatment anxiety and concerns about self‐image, extending from childhood and adolescence into adulthood and impeding better self‐management and impacting them psychosocially.

### 3.3 Limited Use of Pharmacy for Health Advice

This theme illustrates how participants accessed CP support in their self‐management and how CPs' evolving role in the UK towards being prescribers will affect young adults' AD self‐management.

Despite perceiving CPs as helpful because of the knowledge CPs were perceived to have about AD, many participants said they had never gone to CPs for advice and they did not know CPs could offer advice about AD.

‘Pharmacists were out the picture I would say, because back then I didn't know like [CPs] had such a role like in advising patients’.

P3 (female, 20‐year‐old, moderate AD)

Some participants had been in community pharmacies to collect their prescriptions and get over‐the‐counter medicines, but not advice. Participants said they would consider seeing CPs for mild AD, but were doubtful if CPs could manage more severe cases. One participant thought CPs were less specialised in advice for AD than other HCPs, such as dermatologists.

‘I feel like advice wise that [CPs] are limited. Obviously can be specialist in some areas, but with regard to like. . if I was to explain my condition to a [CP], I don't feel like I'd get the best out of it’.

P5 (male, 20‐year‐old, moderate AD)

Participants also felt that by now, they knew how to self‐manage AD, so advice from CPs was not needed. Some participants would share their problems and concerns with their friends or if their AD worsened, participants would primarily consider visiting a general practitioner (GP) or dermatologist. Several participants who had not been brought up in the UK shared a cultural tendency to see a specialist first. They reported they had developed rapport with specialists and continued to consult their specialists, not CPs, because of this.

‘I don't think there were any [HCPs] else because we had a good dermatologist that my mom started going to when I was like a little kid. So [the dermatologist] knows me, my whole life so we are going to see the same [dermatologist] like for years’.

P21 (female, 19‐year‐old, moderate AD)

Most participants said that if CPs were able to prescribe treatments for AD, they would be more likely to see CPs for their self‐management queries. Participants perceived this as a positive step to improve timely access to healthcare.

‘Now you have to wait to get a GP appointment and sometimes it takes really long to get them, but if [CPs] are able to prescribe medicines, it will be a lot easier and faster…’

P4 (female, 20‐year‐old, severe AD)

Participants believed CPs had sufficient knowledge to prescribe for AD treatments and trusted that knowledge. Their perception was that CPs had gone through as comprehensive education as doctors. This was in contrast to previous reports that CPs would only be able to manage mild AD, suggesting a focus on access to treatments as prescribers rather than advice.

‘I don't think [CPs] are any less knowledgeable really. When it comes to prescribing. . yeah I'd trust them as much as the doctor’.

P23 (male, 20‐year‐old, moderate AD)

Participants reported awareness of CPs' knowledge and skills should be raised in young adults because they lacked awareness of CPs' skills.

‘If I'd had like my first breakout of [AD] with knowledge I know now, I could have gone to [CPs] first’.

P2 (female, 20‐year‐old, moderate AD)

Collectively this theme demonstrates participants did not recognise community pharmacy as a source of health advice as they transitioned into self‐managing their AD. Participants did recognise the potential for CPs to support AD self‐management, especially if CPs had the ability to prescribe and maintain access to treatments.

## 4 Discussion

This study found that for many participants, the transition to self‐management improved AD symptoms. Some young adults found that the responsibility for AD self‐management was a challenge, describing controlling their AD as a burden, which affected their mental health and worsened AD. Young adults acknowledged taking responsibility for their AD management as a process of learning. Nevertheless, AD symptoms, especially when visible, affected young adults' mental well‐being and self‐confidence during the transition process. How treatments are used was also impacted by concerns, particularly around TCs originating from social media. Young adults would consider consulting HCPs, but not usually CPs. However, young adults reported that seeking support from CPs who were prescribers, could improve challenges accessing treatment. These findings indicate during the transition from paediatric to adult care, young adults with AD face unique challenges. Further support is needed to prioritise well‐being, navigating concerns about treatments and supporting access to reliable information sources.

Participants highlighted the transition to self‐management as a learning process. To improve AD symptoms, they sought reliable information and recognising the importance of treatment adherence, yet they shared concerns about TC withdrawal syndrome. Fears about TC withdrawal align with existing research with behavioural interventions to seen as a way to challenge this [28]. The concern about withdrawal originated from social media and caused participants to use TCs sparingly. This may impact young adults' treatment adherence and outcomes. In addition, participants were unaware of the FTU as a measure of application, where one FTU from the fingertip to the crease should be enough to cover a full hand [29]. Some participants either underused or overused TCs. Similarly, young adults aged 20–29 were shown as the most affected group by TC misuse [30]. Hence, our findings suggest young adults may struggle to reliably access and interpret clear guidance and information about using medicines to successfully self‐manage AD. Further support should be provided to young adults to access reliable and clear guidance about their AD treatments.

The study found concerns relating to self‐image, particularly on the face, which impacted self‐confidence and persisted from adolescence into adulthood. This finding aligns to a secondary qualitative analysis study in which adolescents and young adults with AD felt different from others within their social networks due to visible symptoms [31]. In addition, previous research highlighted increased anxiety when AD flares on the neck and face [32]. As AD patients' negative perceptions about their visual appearance correlates with reduced mental health [33], practitioners should address young adults' concerns about their appearance, and prioritise the management of visible AD symptoms, contributing to mental well‐being and AD management [32]. As concerns about appearance also caused some participants to overuse TCs on their face, it is essential to address young adults' concerns about appearance not to compromise their mental well‐being and AD self‐management.

Young adults with AD reported limited access to specialist appointments and changes to the availability of medicines resulting in treatment discontinuation [8, 34]. Access to treatment was identified as one of the top 10 priorities for young people with chronic conditions including AD [35]. This is important as newly qualified CPs in the UK will be independent prescribers from 2026 and will be able to offer prescribable treatments without need for AD patients to access specialists or GP for a prescription [36]. Currently, in the UK, CPs are unable to prescribe treatments for AD unless they have an additional independent prescribing qualification. However, existing work shows that GPs faced difficulty referring patients with skin conditions to use pharmacy services [19]. As the participants stated, this may change if pharmacists can prescribe a wider range of treatments, which would help young adults with AD access timely healthcare through CPs.

Although having prescribing CPs would make young adults more likely to seek CP help for AD self‐management, participants also reported limited engagement with CPs to obtain advice. Similarly, according to previous research, HCPs think that young adults look for help from nurses and doctors, not from pharmacists [37]. This highlights that CPs' expertise should be made more widely known to young adults, which would enable CPs to help address young adults' concerns about treatment anxiety related to AD. A study looking at how pharmacists support young people with chronic conditions suggests that as pharmacists may be a valuable source of health advice for young people through their transition to independence, pharmacists should cultivate interest in developing trusting relationships with young people with chronic diseases [38]. If improved relationship is developed between CPs and young adults, this may help young adults become aware of CPs' skills and services they can seek advice for managing AD.

A strength of this study was participants were recruited across a variety of ethnicities, onset of AD and severities. There was diversity regarding gender in participants, but most (*n* = 20) were female. Most participants were currently students and thus relatively educated. Therefore, the findings may be less applicable to young adults who entered into the workforce after leaving compulsory education instead of higher and further education. Most participants had moderate AD, so the findings may not be fully transferable to young adults with severe or mild AD.

## 5 Conclusion

To conclude, despite describing improvements in AD symptoms after transitioning to self‐management, young adults experience unique concerns about their appearance, treatment anxiety, and emotional, environmental, and economic challenges. CPs could help to address young adults' concerns around treatment and AD management as young adults learn to manage AD themselves. This is particularly important given new prescribing status of CPs who may provide products and services to young adults seeking to manage their AD symptoms, but currently CPs are not perceived as a source of support by young adults. Therefore, more work is needed by practitioners and policymakers to improve young adults' awareness of accessing CP support and how this can effectively enhance AD self‐management without compromising young adults' safety.

## Author Contributions

**Abdi Berk Cayci:** conceptualization, data curation, formal analysis, funding acquisition, investigation, project administration, resources, validation, visualization, writing – original draft, writing – review and editing. **Adam Pattison Rathbone:** conceptualization, formal analysis, supervision, validation, visualization, writing – review and editing. **Charlotte Lucy Richardson:** conceptualization, validation, visualization, writing – review and editing, formal analysis, supervision. **Laura Lindsey:** conceptualization, formal analysis, resources, supervision, validation, visualization, writing – review and editing.

## Ethics Statement

Ethical approval was granted by Newcastle University Ethics Committee (Reference number: 22623/2022).

## Conflicts of Interest

The authors declare no conflicts of interest.

## Supporting information

Supplementary material interview guide.

Appendix Standards for Reporting Qualitative Research.

## Data Availability

Representative quotes were used in this article. All data underlying this study cannot be shared publicly because of the privacy of participants.
