# Supplementary material for: Exploring Young Adults' Experiences of Atopic Dermatitis Self‐Management and Use of Community Pharmacy: A Qualitative Study
Source: Health Expect. 2025 Aug 7;28(4):e70378. doi: 10.1111/hex.70378 (PMC12329238; doi:10.1111/hex.70378)
Supplement: Supplementary file 1 — Supplementary material interview guide. [file HEX-28-e70378-s001.docx]

**Interview guide for young adults with atopic dermatitis**

Thank you for agreeing to take part. Before the start, I want to inform you this recording will be deleted after being transcribed, and your name will be anonymized in any part. Is that okey to start?

If you’ve had a look at the information sheet I sent you, I mentioned about Atopic dermatitis (AD) there, which is also called eczema. From now, I will refer Atopic Dermatitis as eczema during the interview.

**Introduction**

1. Tell me about yourself. (Where are you from? How old are you? Do you live alone, with your family or friends, or other?)
2. Could you tell me how long you have had eczema?

(**Prompt:** When did you get diagnosed? **Prompt:** Diagnosis in infancy, childhood, adolescence, or recent past?, **Prompt:** who diagnosed it? By GP, dermatologist, or?)

**Diagnosis**

1. Could you tell me what happened when you were diagnosed with eczema?
   1. **Prompt:** How did you feel then?
   2. **Prompt:** What was happening in your life at that time?
2. How did eczema diagnosis impact you then?
   1. **Prompt:** How did the diagnosis affect your life, such as your general life, school life, friend relations?

**Treatment**

1. Could you tell me about how you manage your condition? (For example: What treatments do you use and how ?)
   1. **Prompt 1:** What was it like before using treatment?
   2. **Prompt 2:** How did you feel about that treatment/regimen? (Have they worked to solve your problem?)
   3. **Prompt 3: Have you had any concern about your treatments so far?**
   4. How did you find out about them? (Were you prescribed or did you find out by yourself?)
   5. What do you think about the effect of social media on your management? (**Prompt:** Have you ever benefited from any social media platform (instagram, twitter, tiktok, etc.) for your management?
   6. Do you just use medications? Is there any other approaches, such as lifestyle habits, to relive your eczema?
2. Tell me who was involved in that management?
3. **Prompt 1:** Did you apply yourself? or did you get any support or help?
4. (If available) How has that support affected your management?
5. Tell me people who are supportive in your treatment nowadays.
   1. How do you think that support (if available) has an impact on you?
6. Tell me if you have ever had any unsupportive comments or reactions from anyone regarding your skin condition?
   1. **Prompt 1:** Have you experienced any stigma?
   2. **Prompt 2:** How did you feel then and what happened?

**Experiences with managing AD**

1. Moving on now to how your experience has changed over time. What has it been like managing your eczema before and after you turned 18?
2. **Prompt:** How was it before 18?
3. Was it more severe or mild? Can you elaborate on it?
4. Is there any difference? Could you give an example?
5. How do you feel about that change?
6. According to the answer they give regarding living style:
7. How has moving out/still living with your family changed your experience?
   - 1. **Prompt**: Could you tell me if moving out home or staying with your family has impacted your eczema experience and management? If so how?

**Interaction with community pharmacists**

1. Which professional groups were involved in your management?
2. What do you think about community pharmacies?
   1. **Prompt:** How do you think community pharmacists contribute to your own management?
3. Do you think community pharmacists are able to support your care?
4. **Prompt 1:** Why?
5. **Prompt 2:** How might community pharmacists be able to help you?
6. **Prompt 3:** Were you given any advice by community pharmacists? If so what and how?
7. **Prompt 4:** What sort of help would you want from pharmacies related to your eczema?
8. Pharmacists in the UK will be able to prescribe in the future. How do you think this will affect your care?
9. Do you think pharmacists are able to prescribe well, do you think they have the competency to prescribe? **Prompt 1:** Why?

**Closing question**

1. Tell me how you would define your experience of managing eczema as a young person with three words.

a. Why did you choose these words?

16) If you had a magic wand, which problem relating to eczema would you want to solve?

a. Why would you want to solve that problem? Could you elaborate on it?
