# Supplementary material for: Exploring Young Adults' Experiences of Atopic Dermatitis Self‐Management and Use of Community Pharmacy: A Qualitative Study
Source: Health Expect. 2025 Aug 7;28(4):e70378. doi: 10.1111/hex.70378 (PMC12329238; doi:10.1111/hex.70378)
Supplement: Supplementary file 2 — Appendix Standards for Reporting Qualitative Research. [file HEX-28-e70378-s002.docx]

**Appendix.** Standards for Reporting Qualitative Research^1^

**Title and abstract Page and line numbers in manuscript**

| **Title** (Concise description of the nature and topic of the study identifying the study as qualitative or indicating the approach (e.g., ethnography, grounded theory) or data collection methods (e.g., interview, focus group) is recommended) | - Page 1 |
| --- | --- |
| **Abstract** (Summary of key elements of the study using the abstract format of the intended publication; typically includes background, purpose, methods, results, and conclusions) | - Page 1-2 |

**Introduction**

| **Problem formulation** (Description and significance of the  problem/phenomenon studied; review of relevant theory and empirical work; problem statement) | - Page 3-4, Lines 1-41 |
| --- | --- |
| **Purpose or research question** (Purpose of the study and specific objectives or questions) | - Page 4, Lines 39-41 |

**Methods**

| **Qualitative approach and research paradigm** (Qualitative approach (e.g., ethnography, grounded theory, case study, phenomenology, narrative research) and guiding theory if appropriate; identifying the research paradigm (e.g., postpositivist, constructivist/ interpretivist) is also recommended; rationale) | - Page 6, Lines 81-82 |
| --- | --- |
| **Researcher characteristics and reflexivity** (Researchers’ characteristics that may influence the research, including personal attributes,  qualifications/experience, relationship with participants, assumptions, and/or presuppositions; potential or actual interaction between researchers’ characteristics and the research questions, approach, methods, results, and/or transferability) | - Page 5, Lines 57-68 |
| **Context** (Setting/site and salient contextual factors; rationale) | - Page 5, Lines 48-50 |
| **Sampling strategy** (How and why research participants, documents, or events were selected; criteria for deciding when no further sampling was necessary (e.g., sampling saturation); rationale^2^) | - Page 4-5, Lines 46-52 - Page 6, Lines 90-92 |
| **Ethical issues pertaining to human subjects** (Documentation of approval by an appropriate ethics review board and participant consent, or explanation for lack thereof; other confidentiality and data security issues) | - Page 5, Lines 53-54 |
| **Data collection methods** (Types of data collected; details of data collection procedures including (as appropriate) start and stop dates of data collection and analysis, iterative process, triangulation of sources/methods, and modification of procedures in response to evolving study findings; rationale) | - Page 6, Lines 69-79 |
| **Data collection instruments and technologies** (Description of instruments (e.g., interview guides, questionnaires) and devices (e.g., audio recorders) used for data collection; if/how the instrument(s) changed over the course of the study) | - Page 6, Lines 69-79 |
| **Units of study** (Number and relevant characteristics of participants, documents, or events included in the study; level of participation (could be reported in results)) | - Page 7, Line 97 - Table 1 (Demographics of participants) |
| **Data processing** (Methods for processing data prior to and during analysis, including transcription, data entry, data management and security, verification of data integrity, data coding, and anonymization/de-identification of excerpts) | - Page 6, Lines 77-79 |
| **Data analysis** (Process by which inferences, themes, etc., were identified and developed, including the researchers involved in data analysis; usually references a specific paradigm or approach; rationale) | - Page 6-7, Lines 80-95 |
| **Techniques to enhance trustworthiness** (Techniques to enhance trustworthiness and credibility of data analysis (e.g., member checking, audit trail, triangulation); rationale) | - Page 6, Lines 86-89 |

**Results/findings**

| **Synthesis and interpretation** (Main findings (e.g., interpretations, inferences, and themes); might include development of a theory or model, or integration with prior research or theory) | - from Page 7, Line 96 to Page 15, Line 256 |
| --- | --- |
| **Links to empirical data** (Evidence (e.g., quotes, field notes, text excerpts, photographs) to substantiate analytic findings) | - from Page 7, Line 96 to Page 15, Line 256 |

**Discussion**

| **Integration with prior work, implications, transferability, and contribution(s) to the field** (Short summary of main findings; explanation of how findings and conclusions connect to, support, elaborate on, or challenge conclusions of earlier scholarship; discussion of scope of application/generalizability; identification of unique contribution(s) to scholarship in a discipline or field) | - from Page 15, Line 257 to Page 17, Line 334 |
| --- | --- |
| **Limitations** (Trustworthiness and limitations of findings) | - Page 17, Lines 318-323 |

References:

1. O’Brien BC, Harris IB, Beckman TJ, et al. Standards for Reporting Qualitative Research: A Synthesis of Recommendations. *Acad Med.* 2014;89(9):1245-51.
